# Simulation of a High-Performance Polarization Beam Splitter Assisted by Two-Dimensional Metamaterials

**DOI:** 10.3390/nano12111852

**Published:** 2022-05-28

**Authors:** Ruei-Jan Chang, Chia-Chien Huang

**Affiliations:** 1 Department of Physics, National Chung Hsing University, Taichung City 40227, Taiwan; grunt230@yahoo.com.tw; 2 Institute of Nanoscience, National Chung Hsing University, Taichung City 40227, Taiwan

**Keywords:** polarization beam splitters, subwavelength gratings, biaxial anisotropy, metamaterials

## Abstract

It is challenging to simultaneously consider device dimension, polarization extinction ratio (PER), insertion loss (IL), and operable bandwidth (BW) to design a polarization beam splitter (PBS) that is extensively used in photonic integrated circuits. The function of a PBS is to separate polarizations of light, doubling the transmission bandwidth in optical communication systems. In this work, we report a high-performance PBS comprising two-dimensional subwavelength grating metamaterials (2D SWGMs) between slot waveguides. The 2D SWGMs exhibited biaxial permittivity by tailoring the material anisotropy. The proposed PBS showed PERs of 26.8 and 26.4 dB for TE and TM modes, respectively, and ILs of ~0.25 dB for both modes, with an unprecedented small footprint of 1.35 μm × 2.75 μm working at the wavelength *λ* = 1550 nm. Moreover, the present structure attained satisfactory PERs of >20 dB and ILs of <0.5 dB within an ultrabroad BW of 200 nm.

## 1. Introduction

To accomplish photonic integrated circuits (PICs) [1,2,3,4], a popular material platform is the silicon on insulator (SOI). There are two primary merits of using the SOI platform: matured semiconductor fabrication technology and the high-index contrast of the refractive index, thereby significantly rendering the devices compact. However, the SOI platform’s highly birefringent property leads to significant polarization dependence, which is undesirable for an optical-fiber network. To resolve this problem, polarization management components, including polarization rotators [5,6,7,8], polarizers [9,10,11,12,13], and polarization beam splitters (PBSs) [14,15,16,17,18,19,20], were proposed. Among them, PBSs are the most popular for separating two orthogonal polarizations, the transverse-electric (TE) and the transverse-magnetic (TM), because of effectively using the two polarizations in transmitting optical signals. The overall performance of a PBS is evaluated by several criteria, such as device footprint, polarization extinction ratio (PER), insertion loss (IL), operable bandwidth (BW), and fabrication tolerances. Most PBSs are based on the directional coupler (DC) type because they can be flexibly designed by adopting diverse structures (e.g., silicon (Si) strip, plasmonic waveguide, multimodal interference effect, and slot waveguides) depending on the selected priorities of the device size, PER, IL, and BW. The authors in [14] proposed a DC-type PBS on a coupler consisting of Si strips. In [15], the authors reported an asymmetric DC-type PBS comprising a Si strip and a hybrid plasmonic waveguide (HPW). They also used an MMI coupler between a Si strip and an HPW to obtain a device footprint of 1.8 × 2.5 μm^2^ [16]. Yue et al. [17] designed a PBS consisting of horizontal slot waveguides [21,22] to enhance TM mode coupling compared to that of a PBS consisting of an Si-strip coupler [14], remarkably shrinking the coupling length from a few hundreds to a few tens of micrometers. However, the PERs of the TE and TM modes are >20 dB over a narrow range of about 18 nm BW [17]. In experiments, the PBS [17] exhibited PERs of 16.8 for TE mode and 14.1 dB for TM mode [18]. Kim et al. [19] reported a 7.5 μm long PBS assisted by a bridged Si waveguide to achieve high PERs over the full C-band wavelength range. They then reported a PBS using a three-waveguide directional coupler [20], showing extremely high PERs of ~40 dB, but with a coupler length of about 29.4 μm.

In recent years, subwavelength-grating metamaterials (SWGMs) [23,24,25] comprising periodically arranged dielectric strips have been widely adopted to build PICs owing to the maturated development of nanoscale-pattern fabrication technology. SWGMs can be considered to be equivalent anisotropic waveguide structures approximated by effective medium theory (EMT) [26,27], and their permittivity tensors can be flexibly engineered to achieve the desired optical properties. As a result, many applications utilizing tailorable anisotropy SWGMs were reported, including waveguide couplers [28,29], low-loss fiber-to-chip couplers [30,31], spectral-filtering devices [32,33], and polarization management [34,35,36,37,38]. In polarization devices, Halir et al. [34] fabricated a beam splitter based on MMI showing it to be three times more compact and with a lower phase error than that of its conventional counterpart. SWGMs were placed between two strip waveguides by Xu and Xiao [35] to reduce TM mode coupling and increase TE mode coupling. Liu et al. [36] embedded SWGMs in a coupler comprising two strip wires to attain BW broadening by altering the effective index of the even mode. Xu and Shi [37] fabricated an ultrasharp multimode waveguide bending assisted by SWGM mode converters to inhibit intermodal coupling. Adopting tilted SWGMs, Luque-González et al. [38] reported a PBS with 72 nm BW, IL < 1 dB, PER > 15 dB, and device length of 14 μm. 

Instead of locating SWGMs in the waveguide-core region [34,35,36,37,38], some research groups [39,40,41] theoretically proposed and experimentally adopted SWGMs in cladding regions to reduce crosstalk between Si-strip waveguides by engineering the depths of evanescent waves called extreme skin-depth (*e*-skid) waveguides. Li et al. [42] realized a PBS adopting cascaded dual-core tapers. Mao et al. [43] reported a PBS with ultrabroad BW > 420 nm using tapered SWGM and slot waveguides, with ILs < 0.8 dB and PERs > 10.9 dB. Mia et al. [44] reported adiabatically tapered e-skid to form a PBS capable of operating at a large BW of 250 nm; however, the device length of 100 μm led to severe limitations for building densely integrated photonic circuits. Zhang et al. [45] experimentally demonstrated an *e*-skid-based PBS with the performance of BW > 80 nm, PERs > 20 dB, ILs < 1 dB, and 14 μm long device length. Xu et al. [46] discovered a PBS that extended SWGMs in both core and cladding regions. The PBS was designed to function as an MMI coupler for TM mode, but as two isolated waveguides for TE mode. Inspired by the idea [47] of *e*-skid waveguides that are implemented by SWGMs in parallel and perpendicular directions to engineer evanescent wave allowing, we present a PBS consisting of a two-dimensional SWGMs (2D SWGMs) between two slot waveguides to extend the applications of conventional one-dimensional SWGMs (1D SWGMs). The present PBS dramatically reduces (enhances) the mode couplings of TE (TM) mode, thus concurrently achieving an extremely short PBS with high PERs, low ILs, and ultrabroad BW. The dependences of geometrical dimensions on device performance are analyzed in detail. The spectral response was also investigated to show the broadband operation. Lastly, we address fabrication tolerance to validate the robustness of the proposed PBS.

## 2. Mode Characteristic and Coupling Effect with Anisotropic SWGMs

**Figure 1:**
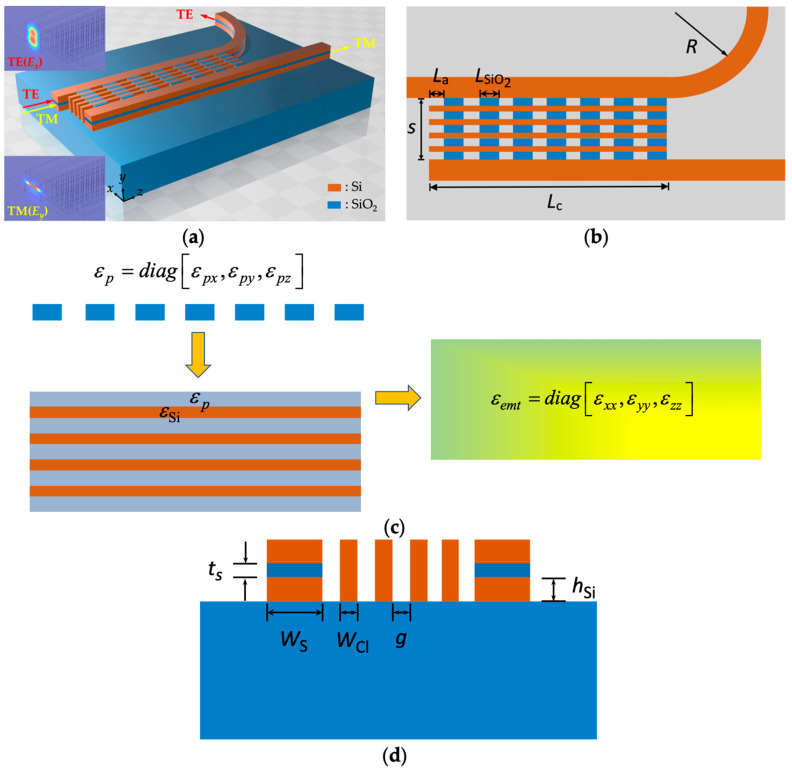
(**a**) 3D diagram with TE (*E_x_*) and TM (*E_y_*) mode profiles in the incident plane; (**b**) top view; (**c**) schematic of calculating the resultant effective permittivity *ε_emt_* of 2D SWGMs, which is obtained by sequentially estimating the 1D SWGMs in the *z* (*ε_p_*) and *x* directions (*ε_emt_*) on the basis of EMT between slot waveguides; (**d**) cross-section in *xy* plane of the present PBS.

Figure 1a–c show the 3D diagram with the TE (*E_x_*) and TM (*E_y_*) mode profiles in the incident plane, the top view, and the cross-section in the *xy* plane, respectively, of the proposed PBS. Slot waveguides on a SiO_2_ substrate comprised a SiO_2_ slot layer sandwiched with high-index Si strips in which the TE channel was connected to a bent waveguide with a radius of curvature (*R*) at the end in order to effectively decouple the two slot waveguides. In the slot waveguides with width *W*_S_, the thicknesses of the slot layers and Si layers were *t_s_* and *h*_Si_, respectively. The height and width of Si strips (copper red) were 2*h*_Si_ + *t_s_* and *W*_S_, respectively. The pitch of SWGMs in the *x* direction was set to *Λ_x_* = *W*_Cl_ + *g* with duty cycle *ρ_x_* = *W*_Cl_ /*Λ_x_*, where *W*_Cl_ is the width of the Si strips, and *g* is the gap between Si strips. Edge-to-edge spacing between slot waveguides is *s*. Likewise, the pitch of SWGMs in the *z* direction was set to be *Λ_z_* = *L_a_* + *L*_SiO2_ with duty cycle *ρ_z_* = *L*_SiO2_/*Λ_z_*, where *L_a_* is the gap between SiO_2_ grating (dull blue).

**Figure 2:**
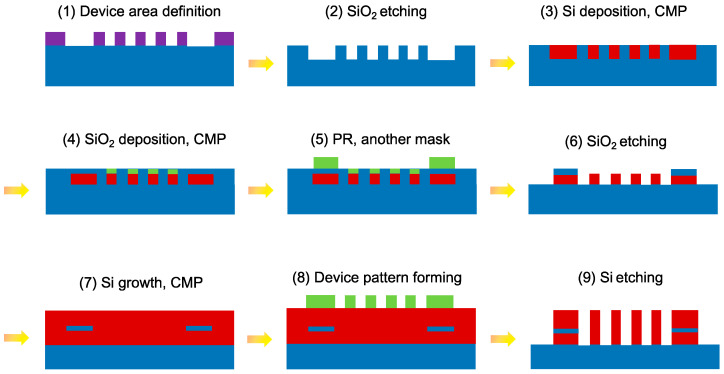
Schematic diagram of the fabrication processes of the designed PBS.

Before analyzing the proposed design, the fabrication processes are schematically illustrated in Figure 2. (1) A negative photoresist (PR) film (purple) was deposited to pattern the lower Si strips with a hard mask defining the patterns of the proposed structure on a SiO_2_ substrate (blue); then PR exposure and development were conducted. (2) The proposed structure was formed by etching SiO_2_ and lifting off the PR film. (3) A Si film was deposited by chemical vapor deposition, and the Si layer was planarized by chemical mechanical polishing (CMP). (4) Depositing a positive PR (green) to pattern the SWGMs with another mask was followed by PR exposure and development, SiO_2_ slot layer deposition using thermal oxidation, and a CMP was conducted. (5) Similar to Step 4 but with a different mask. (6) After SiO_2_ reactive ion etching, the PR film was lifted off. (7) The upper Si strips and SWGMs were deposited, and a CMP was carried out. (8) A positive PR was deposited with the same mask as that in Step 1, followed by PR exposure and development. (9) After Si etching, the positive PR film was lifted off to reach the desired structure. Lastly, the SiO_2_ regions between the SWGMs could be formed with the procedures of SiO_2_ deposition, PR deposition, patterning the 2D SWGMs with a mask, SiO_2_ etching, and lifting off the PR.

The working principle of the present PBS is that we propagated TE mode with major electric field *E_x_* along the bar, while TM mode was coupled with a major electric field *E_y_* under the phase matching condition (PMC) into the cross, as shown in Figure 1a. Using the EMT [26,27], the proposed 2D SWGMs displayed an equivalent material anisotropy *ε_emt_* by considering the permittivity of Si, *ε*_Si_ and *ε_p_* as follows (see Figure 1c):

(1) $$\varepsilon_{emt}^{}=diag\left[\varepsilon_{xx}^{},\varepsilon_{yy}^{},\varepsilon_{zz}^{}\right]$$

(2) $$\varepsilon_{xx}^{-1}=\rho_{x}\varepsilon_{\mathrm{Si}}^{-1}+(1-\rho_{x})\varepsilon_{px}^{-1},$$

(3) $$\varepsilon_{yy}^{}=\rho_{x}\varepsilon_{\mathrm{Si}}^{}+\left(1-\rho_{x}\right)\varepsilon_{py}^{},$$

(4) $$\varepsilon_{zz}^{}=\rho_{x}\varepsilon_{\mathrm{Si}}^{}+\left(1-\rho_{x}\right)\varepsilon_{pz}^{},$$

where *ε_xx_*, *ε_yy_*, and *ε_zz_* denote permittivity in the *x*, *y*, and *z* directions, respectively. In this work, *ε_p_* = *diag* [*ε_px_*, *ε_py_*, *ε_pz_*] denotes the equivalent anisotropic permittivity of the SWGMs consisting of SiO_2_ strips and air gaps along the *z* direction, as shown below:

(5) $$\varepsilon_{px}^{}=\varepsilon_{py}^{}=\rho_{z}\varepsilon_{{\mathrm{SiO}}_{2}}^{}+(1-\rho_{z})\varepsilon_{\mathrm{air}}^{},$$

(6) $$\varepsilon_{pz}^{-1}=\rho_{z}\varepsilon_{{\mathrm{SiO}}_{2}}^{-1}+(1-\rho_{z})\varepsilon_{\mathrm{air}}^{-1},$$

where *ε*_SiO_2__ and *ε*_air_ denote the permittivity of SiO_2_ and air, respectively. Differing from the previous reports [24,25,26,27,28,29,30,31,32,33,34,35,36,37,38,39] using one-dimensional SWGMs, *ε_emt_* in Equation (1) showed biaxial anisotropy, thus more flexibly tuning the optical characteristics. To design a PBS here, the mode characteristics of the TE and TM modes had to be obtained in advance. After that, the coupling length of a coupled waveguide for a specific mode measuring the distance required to completely transfer power from one waveguide to another can be computed by *L_c,i_* = *λ*/{*2*(*n_i,even_* − *n_i,odd_*)} [48], where *i* denotes TE or TM. *n_i,even_* and *n_i,odd_* denote the effective indices of the even and odd modes, respectively. 

**Figure 3:**
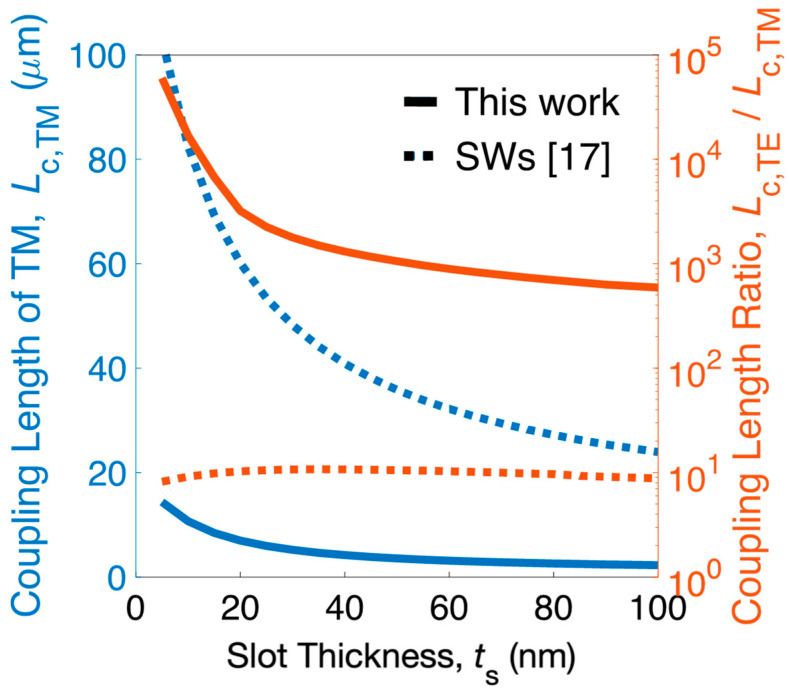
(left axis) Coupling length of TM mode *L_c,_*_TM TM_ and (right axis) coupling-length ratio *L*_c,TE_/*L*_c,_ versus *t*_s_ for the present PBS and the SWs.

First, we analyzed the mode characteristics of the present design. The relative permittivity of Si and SiO_2_ was *ε*_Si_ = 12.110 and *ε*_SiO_2__ = 2.085 [49], respectively, at *λ* = 1550 nm. The chosen parameters were *W*_Si_ = 400 nm, *W*_Cl_ = 75 nm, *g* = 50 nm (i.e., *ρ_x_* = 0.6), *h*_Si_ = 150 nm, *L*_a_ = 100 nm, *L*_SiO__2_ = 150 nm (i.e., *ρ_z_* = 0.6), *s* = 550 nm, and four Si strips between slot waveguides. First, we obtained the anisotropic *ε_p_* = *diag* [1.285, 1.285, 1.206] according to Equations (5) and (6). Next, the resultant biaxially anisotropic *ε_emt_* = *diag* [1.851, 2.815, 2.805] could be obtained according to Equations (2)–(4). With the obtained *ε_emt_* in the region between slot waveguides and using COMSOL Multiphysics 6.0 (COMSOL Inc., Burlington,VT, USA), the coupling length of TM mode *L*_c,TM_ and the coupling-length ratio of TE and TM modes *L_c,_*_TE_/*L*_c,TM_ versus *t*_s_ for the present PBS and the conventional SWs [17,18] are shown in Figure 3. The larger value of *L_c,_*_TE_/*L*_c,TM_ implies that more TE power is retained in the bar while TM power is completely transferred to the cross. *L_c,_*_TE_ was far larger than *L*_c,TM_ due to better TE mode confinement; thus, a PBS’s length is determined by a shorter *L*_c,TM_. For conventional SWs [17], *L*_c,TM_ (dull blue dotted line) varied from 82.42 to 32.19 μm for *t*_s_ for 10 and 60 nm, respectively, and *L_c,_*_TE_/*L*_c,TM_ (copper red dotted line) was only 9.16 at *t*_s_ = 60 nm. In contrast, the proposed structure not only shrunk *L*_c,TM_ (dull blue solid line) to 10.69 and 3.15 μm for *t*_s_ = 10 and 60 nm, respectively, but also improved *L_c,_*_TE_/*L*_c,TM_ (copper red solid line) to 891 at *t*_s_ = 60 nm. Results demonstrate that the device length and PER of a PBS based on a two-SW can be significantly reduced and improved, respectively, by locating the proposed 2D SWGMs between the two SWs.

**Figure 4:**
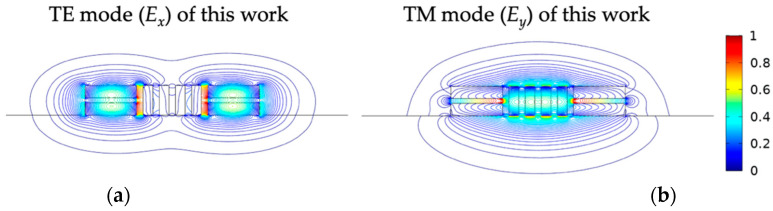

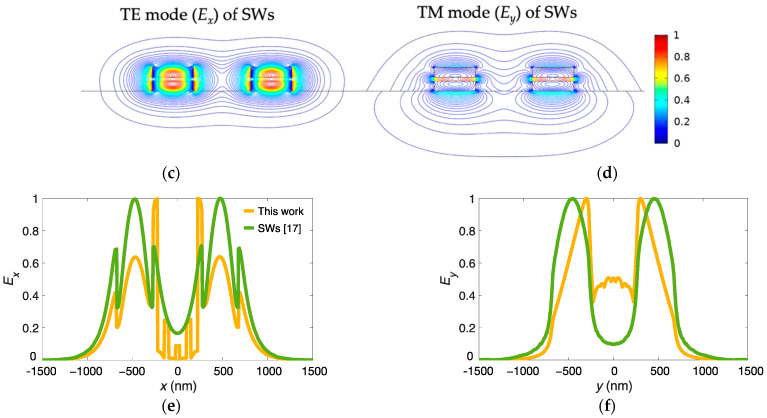
Field contours of the even modes of (**a**) TE and (**b**) TM and those of (**c**) TE and (**d**) TM of the SWs for *W*_Si_ = 400 nm, *t*_s_ = 60 nm, *h*_Si_ = 150 nm, *W*_Cl_ = 75 nm, *g* = 50 nm, *s* = 550 nm, and *λ* = 1550 nm. (**e**) Field amplitudes at the central lines of the slot along the *x* directions of (**a**,**c**); (**f**) field amplitudes at the central lines of the slot along the *x* directions of (**b**,**d**).

Further 3D calculations of mode and propagation characteristics are outlined in the next section. For observing the mode coupling, the even TE and TM field contours of the present structure are exhibited in Figure 4a,b, respectively, and those of the SWs are exhibited in Figure 4c,d, respectively.

By adding 2D SWGs in the middle region of conventional SWs, the decaying rates of evanescent wave of the TE and TM modes are considerably suppressed (i.e., larger *L_c,_*_TE_/*L*_c,TM_) and enhanced (i.e., shorter *L*_c,TM_), respectively. In addition, the relative field amplitudes of the TE and TM modes along the line denoted in the inset of Figure 4e are shown in Figure 4e,f, respectively. The above results can be explained by the decay constant of the TE mode *k*_TE_ that can be adjusted by the *ε_zz__/_ε_xx_* ratio according to the dispersion relation [39], and the *ε_zz_* > *ε_xx_* condition is always fulfilled. Therefore, 2D SWGMs significantly reduce crosstalk compared to an isotropic cladding with *ε_zz/_ε_xx_* = 1. In contrast, the decay constant of the TM mode, *k*_TM_, depends on the *ε_zz/_ε_yy_* < 1 ratio, leading to weaker mode confinement.

## 3. Performance Dependences on Duty Cycle, Wavelength, and Fabrication Tolerance

**Figure 5:**
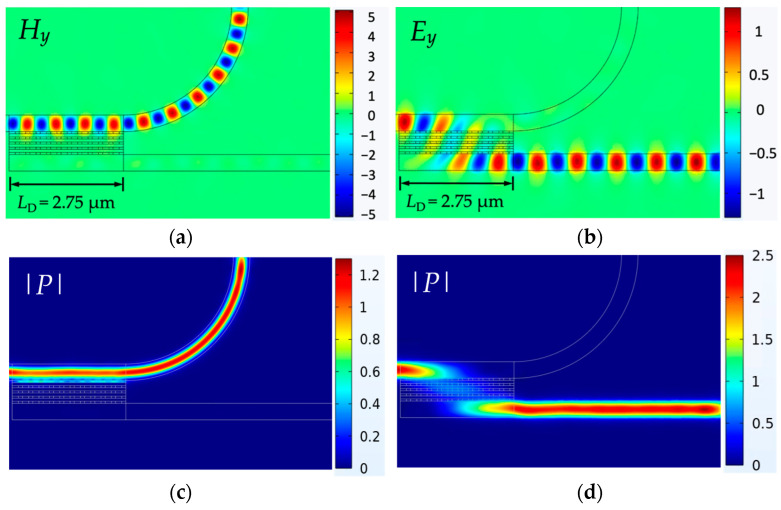
*y* components of (**a**) magnetic field of the TE mode and (**b**) electric field of the TM mode, and the total power evolutions of the (**c**) TE and (**d**) TM modes of the present structure with device length *L*_D_ = 2.75 μm (i.e., the practical *L*_c,TM_ with optimal performance) for *t*_s_ = 60 nm, *h*_Si_ = 150 nm, *W*_Cl_ = 75 nm, *W*_Si_ = 400 nm, *g* = 50 nm, *s* = 550 nm, and *λ* = 1550 nm.

Device performance dependences on geometry parameters, wavelength response, and fabrication tolerance proceeded by executing 3D simulations. To assess the transmission performance of a PBS, we analyze the PERs and ILs of both modes, which are formulated in Equations (7) and (8), respectively:

(7) $${\mathrm{PER}}_{TE(TM)}=10\;\log_{10}\left(\frac{P_{TE(TM),\;bar(cro)}}{P_{TE(TM),\;cro(bar)}}\right),$$

(8) $${\mathrm{IL}}_{TE(TM)}=\;-10\;\log_{10}\left(\frac{P_{TE(TM),\;bar(cro)}}{P_{in}}\right),$$

where *P_in_* denotes the input power, *P_TE_*_(*TM*)*,bar*(*cro*)_ denotes the TE (TM) mode power at the bar (cross), and *P_TE_*_(*TM*)*,cro*(*bar*)_ is the TE (TM) mode power at the cross (bar). By adopting *R* = 3 μm and the same parameters displayed in Figure 4, the *y* component of the magnetic field (*H_y_*) and total power (|*P*|) evolutions of TE mode are shown in Figure 5a,c, respectively, and the *y* component of the electric field (*E_y_*) and total power (|*P*|) evolutions of TM mode are shown in Figure 5b,d, respectively.

**Figure 6:**
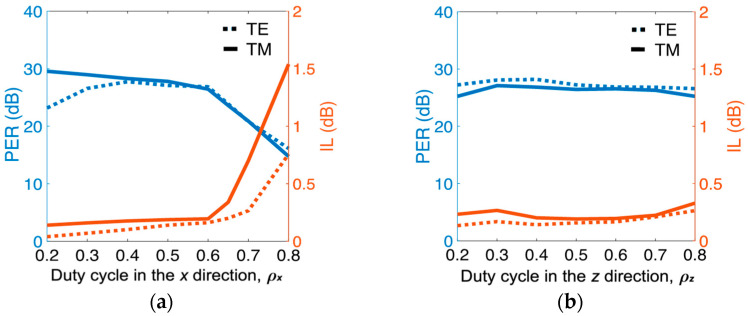
(left axis) PER and (right axis) IL of both modes as functions of duty cycles (**a**) *ρ*_x_ and (**b**) *ρ*_z_ at the same parameters as those in Figure 4.

In Section 2, the calculated *L*_c,TM_ at *t_s_* = 60 nm was 3.15 μm on the basis of equivalent permittivity *ε_emt_*. The practical *L*_c,TM_ to obtain optimal performance is device length *L*_D_ = 2.75 μm, which was shorter than the calculated *L*_c,TM_ in Section 2 because the coupling continued a short distance from the entrance of the bent waveguide and then gradually decreased. At device length *L*_D_ = 2.75 μm, the obtained results were PER_TE_ = 26.81 dB, PER_TM_ = 26.48 dB, IL_TE_ = 0.16 dB, and IL_TM_ = 0.19 dB. By contrast, conventional SWs [17] with *L*_c,TM_ = 34.6 μm achieved PER_TE_ = 25.5 dB, PER_TM_ = 14.8 dB, IL_TE_ = 0.07 dB, and IL_TM_ = 0.06 dB. Results show that the proposed PBS not only considerably reduced the proposed PBS’s length by about 12 times when compared to the conventional SWs [17], but also achieved superior PERs, particularly for PER_TM_. Although the ILs of the current design were higher than those of the SWs, values of <0.2 dB were still acceptable. Considering the effect of duty cycles, we show the PERs and ILs versus *ρ_x_* in Figure 6a. The optimal PERs and ILs of both modes appeared in the interval of *ρ_x_* = 0.4 to 0.6. Once the condition of *ρ_x_* > 0.6 had been reached, PERs and ILs dramatically degraded because a larger *ρ_x_* decreases (increases) the value of *ε_zz/_ε_xx_* (*ε_zz/_ε_yy_*) leading to smaller (larger) *k*_TE_ (*k*_TM_), simultaneously increasing the crosstalk of both modes. The higher PERs are also reflected in lower ILs. Although optimal PERs and ILs were at *ρ_x_* = 0.4 or 0.5 here, the device length was *L*_D_ = 6.50 or 4.15 μm, respectively. Considering compactness, we chose *ρ_x_* = 0.6 to investigate the subsequent analyses, and PERs and ILs versus *ρ_z_* are exhibited in Figure 6b.

**Figure 7:**
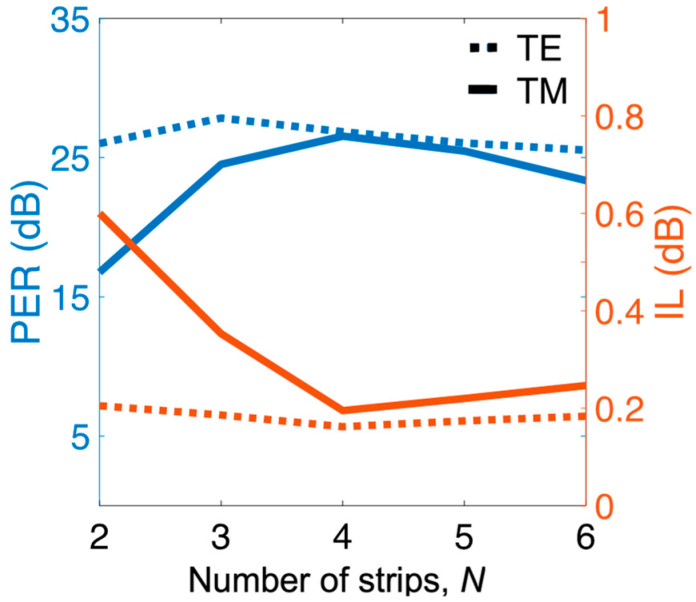
(left axis) PER and (right axis) IL of both modes as a function of the number of Si strips, *N* between slot waveguides at the same parameters as those in Figure 5.

The ILs of the TE and TM modes slightly increased, ranging from *ρ_z_* = 0.2 (*L*_D_ = 2.9 μm) to 0.8 (*L*_D_ = 2.55 μm), and PER_TE_ and PER_TM_ slightly varied as *ρ_z_* varies. Differing from the major effect of *ρ_x_*, *ρ_z_* plays a finetuning role on device performance and length. Regarding to the number of Si strips (*N*), the PERs and ILs versus *N* (at the condition of *ρ_x_* =0.6 and *ρ_z_* = 0.6) are shown in Figure 7. The proposed PBS achieved optimal performance when *N* = 4 was chosen. Further increasing *N* resulted in moderately varying performance. The performance of TM mode dramatically worsened as *N* decreased. This result can be attributed to t *Λ_x_* being increasingly away from the condition of *Λ_x_* << *λ*, causing the increase in scattering loss that resulted from the grating structure [21].

**Figure 8:**
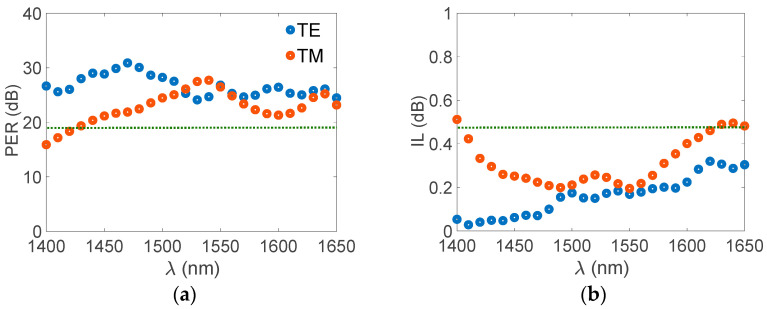
(**a**) PER and (**b**) IL versus wavelength at the same parameters as those in Figure 4.

To assess the BW of the present PBS, the PERs and ILs as a function of wavelength are shown in Figure 8a,b, respectively. PER_TM_ (IL_TM_) significantly decreased (increased) as the wavelength moved away from the target wavelength of *λ* = 1550 nm because the short *L*_c,TM_ was more sensitive than the extremely long *L*_c,TE_ to deviation from the PMC. By contrast, the PER_TE_ and IL_TE_ of the shorter (longer) than *λ* = 1550 nm wavelengths were higher (lower) and lower (higher), respectively. This can be explained by the guided modes with shorter (longer) wavelengths leading to shorter (longer) evanescent wave tails, thus reducing (increasing) crosstalk between waveguides. The working BW of both modes with PERs > 20 dB and ILs < 0.5 dB ranged from *λ* = 1440 to 1650 nm (>200 nm).

To show our design’s superiority, we compared its overall performance with that of SWGM-based PBSs, as shown in Table 1. The footprint of the proposed structure was the smallest PBS compared with the reported PBSs [19,20,42,43,44,45,46], rendering it more beneficial in constructing a highly dense photonic component.

**Figure 9:**
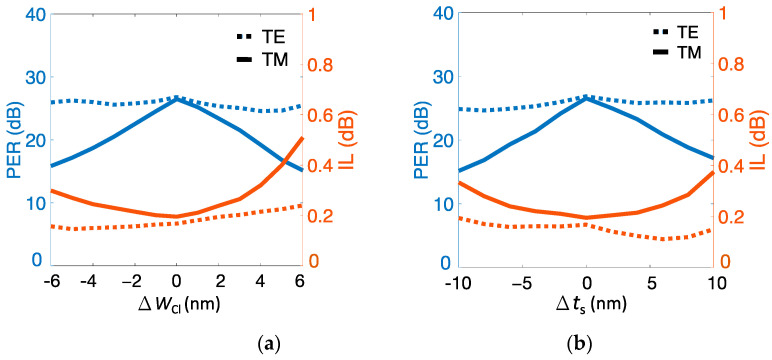
(left axis) PER and (right axis) IL as functions of variations in the (**a**) Si strip width Δ*W*_Cl_ and (**b**) slot thickness Δ*t_s_* of the present structure.

In addition to analyzing performance dependence on duty cycles and number of Si strips, as shown in Figure 6 and Figure 7, respectively, we investigated severe geometries *t*_s_ and *W*_Cl_ to assess fabrication tolerance. Performance versus variations in Si strip width Δ*W*_Cl_ and slot thickness Δ*t*_s_ is shown in Figure 9a,b, respectively. PER_TM_ significantly depended on the two fabrication errors (Δ*W*_Cl_ and Δ*t*_s_) because of the extremely short *L*_c,TM_ = 2.75 μm of the TM mode. As shown in Figure 9a, PER_TM_ decreased to ~17 dB (from ~26 dB), and IL_TM_ increased to ~0.4 dB (from ~0.2 dB) while Δ*W*_Cl_ > 5 nm or Δ*t*_s_ > 10 nm. Within the variation range of Δ*W*_Cl_ < 3 nm or Δ*t*_s_ < 5 nm, PER_TM_ maintained values of >20 dB and IL_TM_ < 0.25 dB. In contrast, PER_TE_ and IL_TE_ showed slight variations in Δ*W*_Cl_ and Δ*t*_s_ due to the exceptionally long *L*_c,TE_ = 891 μm of TE mode.

**Figure 10:**
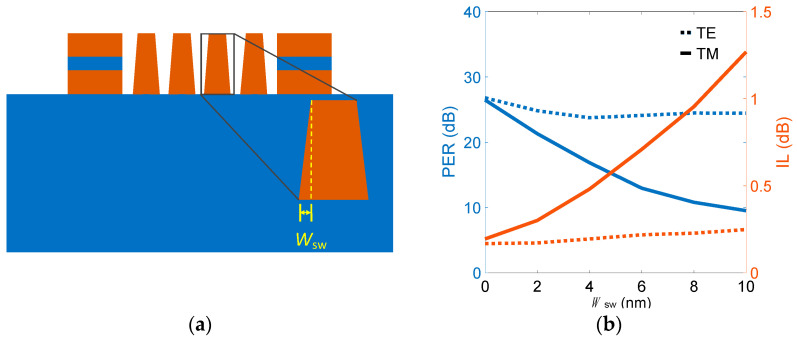
(**a**) Cross-sectional diagram in *xy* plane and zooned-in view of a SWGM, where *W*_sw_ denotes the difference between the bottom and top of the SWGMs. (**b**) PER and IL versus *W*_sw_.

Another essential fabrication error, angled sidewall, frequently occurs in etching processes that typically do not have a perfect 90° sidewall. In the present structure, the crucial parts are SWGMs due to their larger aspect ratio compared to that of the slot waveguides. Therefore, we now discuss the sidewall effect of the SWGMs on device performance. The width difference between the bottom and top of the SWGMs is *W*_sw_, as shown in Figure 10a, and the PERs and ILs of both modes are shown in Figure 10b. PER_TE_ (PER_TM_) and IL_TE_ (IL_TM_) showed slight (significant) dependences on *W*_sw_ due to the extremely weak (strong) coupling strength induced by the 2D SWGMs. PER_TM_ decreased to about 14.2 (9.8) dB, and IL_TM_ increased to about 0.62 (1.25) dB, while *W*_sw_ increased to 5 (10) nm.

## 4. Conclusions

An ultracompact and broadband PBS comprising slot waveguides assisted by in between 2D SWGMs was proposed to increase the integration density and transmission bandwidth of photonic devices. The proposed 2D SWGMs served as both a barrier to suppress TE mode coupling and bridging to improve TM mode coupling by carefully engineering the duty cycles of the SWGMs. The numerical results of the proposed PBS showed PERs of 26.8 and 26.4 dB for TE and TM modes, respectively, and ILs of ~0.25 dB for both modes, with a compact PBS of 1.35 μm × 2.75 μm. Moreover, the present PBS achieved satisfactory performance while operating in a BW of 200 nm. Fabrication tolerance analyses showed that the PER_TM_ maintained superior values of >20 dB and IL_TM_ < 0.25 dB within the variation range of clad width Δ*W*_Cl_ < 3 nm or slot thickness Δ*t*_s_ < 5 nm.

## Figures and Tables

**Table 1:** Performance comparison of SWGM-based PBSs.

Structure	Footprint (μm^2^)	PERs (dB)	ILs (dB)	BW (nm)
[19]	1.7 × 12.25	22.5	1.8	>100
[20]	2.41 × 29.4	39.0	0.35	>100
[42]	2.5 × 33.6	20	0.3	270
[43]	1.3 × 4.6	10.9	0.8	420
[44]	2.75 × 100	20	1.2	250
[45]	2.5 × 14	20	1	80
[46]	1.9 × 12.25	20	1	200
This work	1.35 × 2.75	20	0.5	200

## Data Availability

Data presented in this study are available on request from the corresponding author.
